# Supplementary material for: Improved genetic discovery and fine-mapping resolution through multivariate latent factor analysis of high-dimensional traits
Source: Cell Genom. 2025 Apr 11;5(5):100847. doi: 10.1016/j.xgen.2025.100847 (PMC12143322; doi:10.1016/j.xgen.2025.100847)

Cell Genomics, Volume *5*

Supplemental information

Improved genetic discovery and fine-mapping resolution through multivariate latent factor analysis of high-dimensional traits

Feng Zhou, William J. Astle, Adam S. Butterworth, and Jennifer L. Asimit

Document Supplemental Information: Figures S1-S11.

Figure S1. Selection of the number of latent factors for 99 blood cell traits in 18,310 individuals. Related to STAR Methods. This scree plot illustrates that 25 latent factors, as suggested by Horn's parallel method implemented in the "psych::fa.parallel" function in R, is optimal to explain the variability in 99 blood cell traits; the vertical line is at 25 latent factors. The blue line indicates the eigenvalues calculated in the observed data and the red dashed line corresponds to those calculated in random (noisy) data sets. The selection of 25 factors is the point where the observed data eigenvalues are smaller than those in the random data.

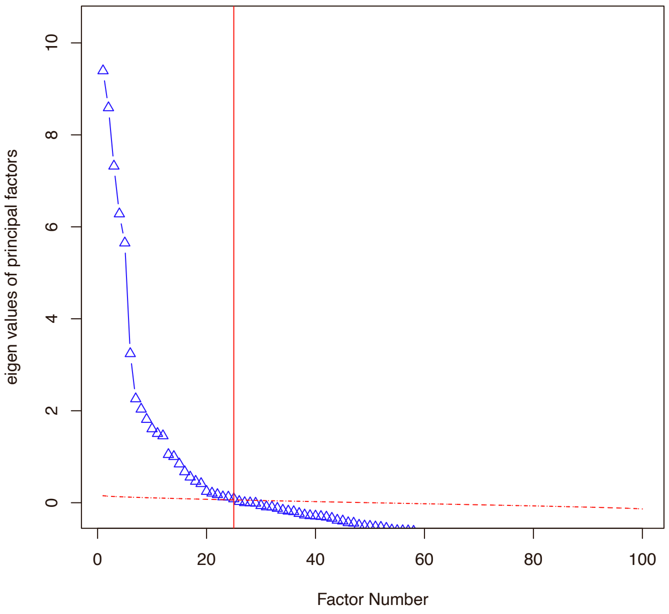

Figure S2. Basophil-related latent trait ML8 is associated with rs9310935, which has moderate signals with multiple basophil traits. Related to Figure 4, STAR Methods. (A) regional association plot, highlighting rs9310935 and conditional regional association plot, conditioned on, rs3217673, rs163546, rs1669340, rs17027750, rs163563, rs3856850, rs334782, rs1695315, rs10212483, rs13097407, rs6787336 which are lead SNPs for basophil-related traits from previous publications (rs163546 (BASO%GRAN), rs3856850/rs10212483/rs163563/rs17027750 (BASO%), rs13097407 (EO%), rs1695315 (EO#), rs334782 (BASO# and BASO-FSC-DW), rs1669340 (BASO-SFL-DW and BASO-FSC-DW), rs3217673 (BASO%, BASO-SFL-DW and BASO-FSC-DW) and rs6787336 ((EO+BASO)#)).

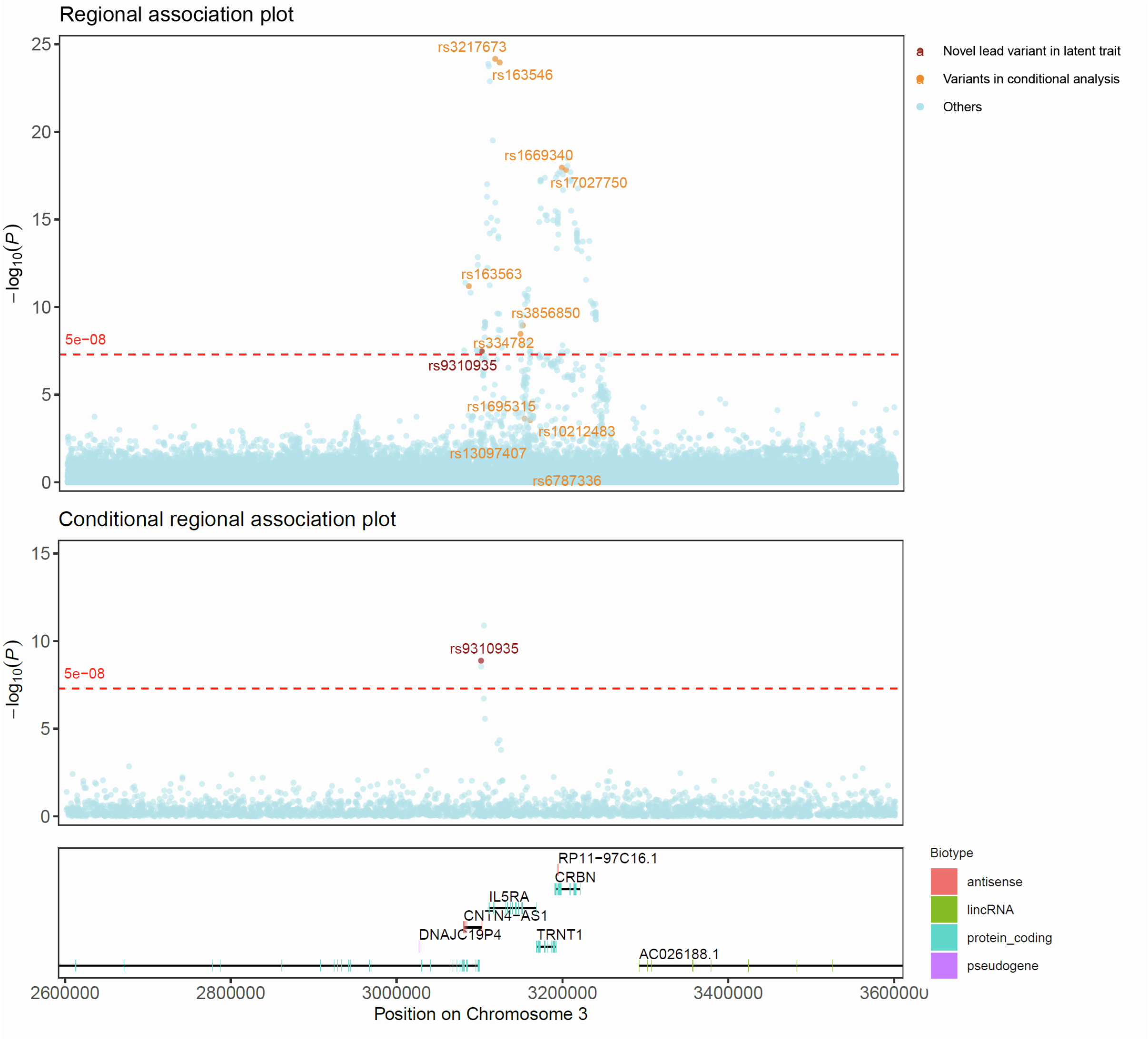

Figure S3. Regional association plots of rs6064377 for ML6 and its related raw traits, HCT, RBC#, HGB, RPI. Related to Table 1, STAR Methods. The top right panel also shows the ML6 association plot, conditioned on previously published lead variants.

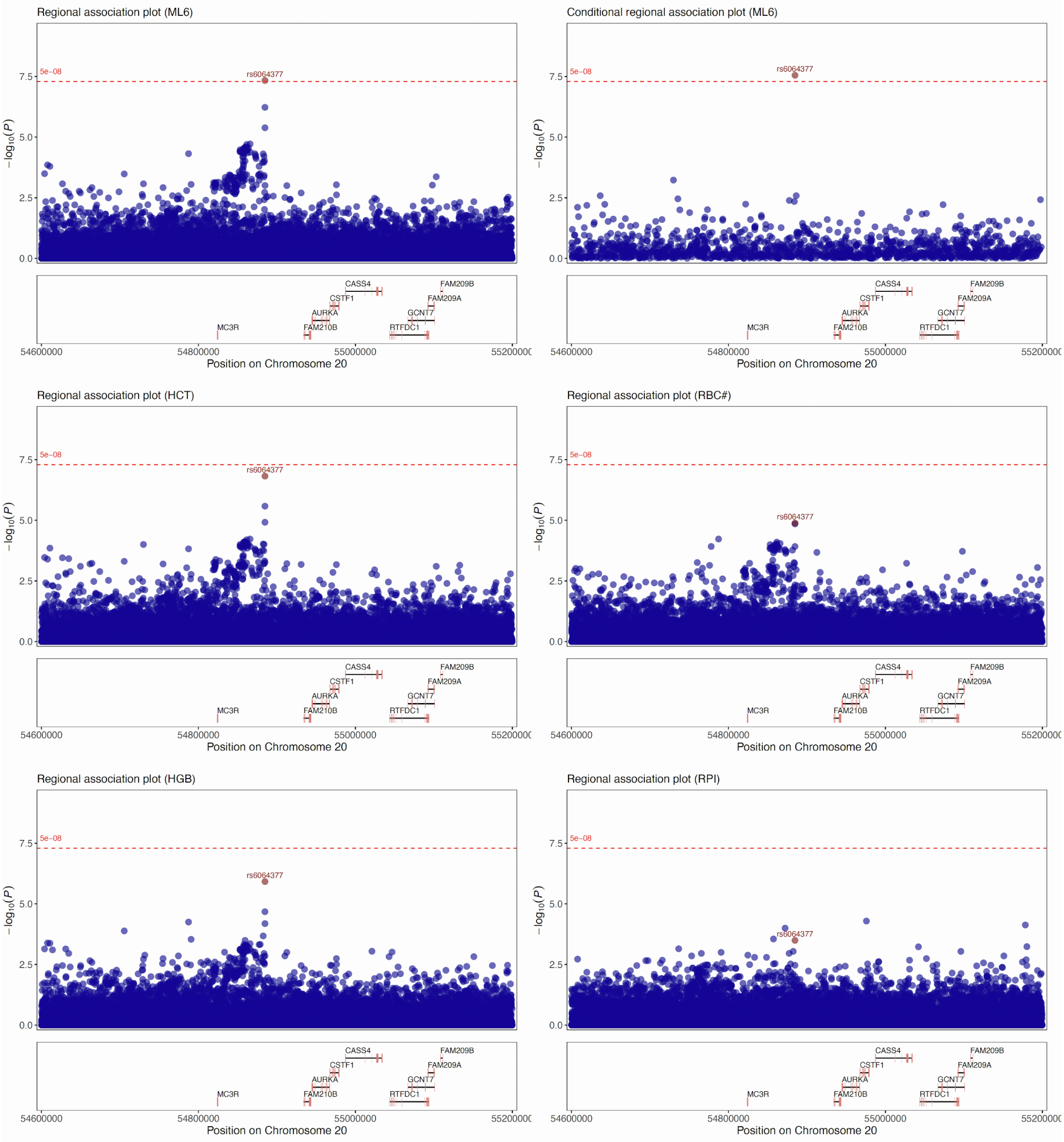

Figure S4. Latent factor interpretations and fine-mapping. Related to STAR Methods. (A) Latent factor contributions (re-scaled factor loadings) facilitate latent factor interpretations upon grouping observed traits with the same maximum-contributing latent factor. **(B)** Fine-mapping of latent factor signals is carried out by single-trait fine-mapping in all regions and by multi-trait fine-mapping when there are at least two latent factors with a signal ($P < 5 \times 10^{-8}$). For each latent factor with a signal, observed traits that have a contribution of at least 20% from the latent factors and that have a signal in the region, are fine-mapped using single-trait fine-mapping. We denote the number of latent factors with a signal by *S* and let *T* be the number of observed traits that satisfy the fine-mapping criteria.

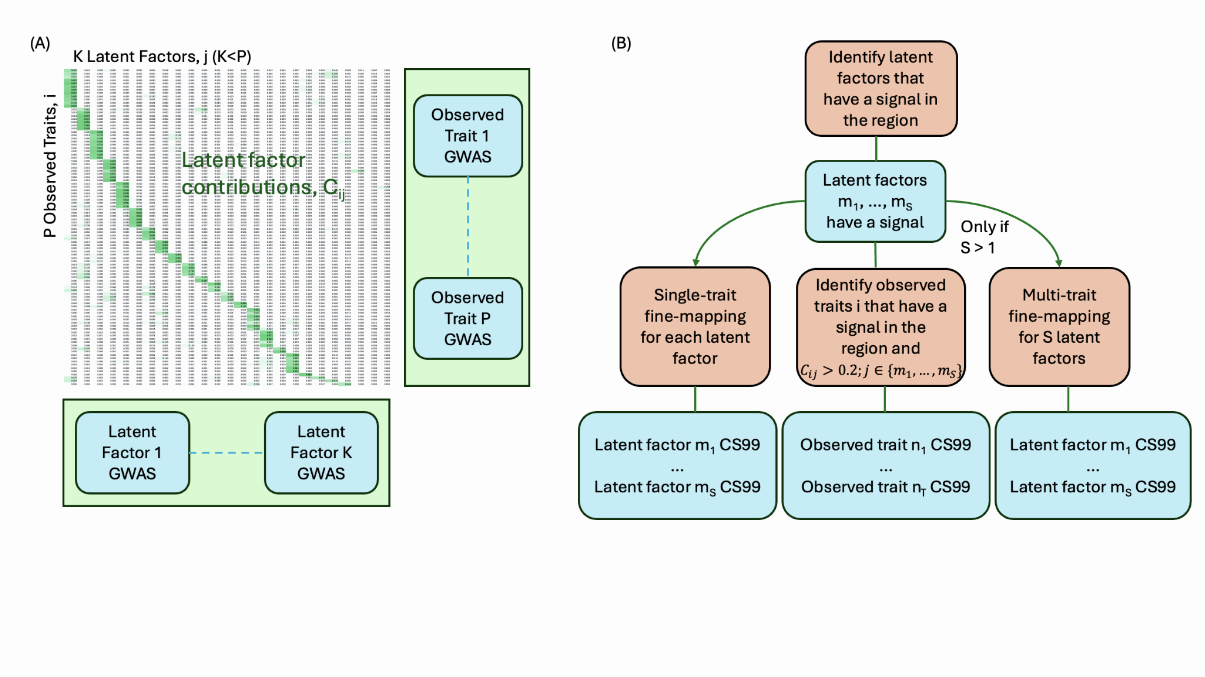

Figure S5. In the *PIEZO1* region, the 99% credible sets (CS99) for raw traits become more refined for latent traits and gain further refinement by latent multi-trait fine-mapping. Related to STAR Methods. Variants belonging to at least one CS99 are listed at the upper axis, labelled rsID. The CS99 for latent traits from single-trait fine-mapping are shown by the latent trait name, e.g. ML12. The CS99 for latent traits from multi-trait fine-mapping are shown by the latent trait name with an asterisk, e.g. ML12*. The coloured dots indicate which variants are members of the CS99 for the listed trait, where the size of each dot is proportional to the marginal posterior probability (MPP) of causality. Dot colours coincide with SNP groups, which are groups of variants in high LD ($r^2 > 0.8$) and with MPP > 0.01 as estimated by the fine-mapping method. Boxes indicate latent and raw traits that are related to each other and the CS99 sizes are provided for each trait, listed by increasing size, e.g. ML12 and ML12* both have a CS99 of size 3 and MCHC has 4 variants in its CS99 - these three traits are related to each other as indicated by the box.

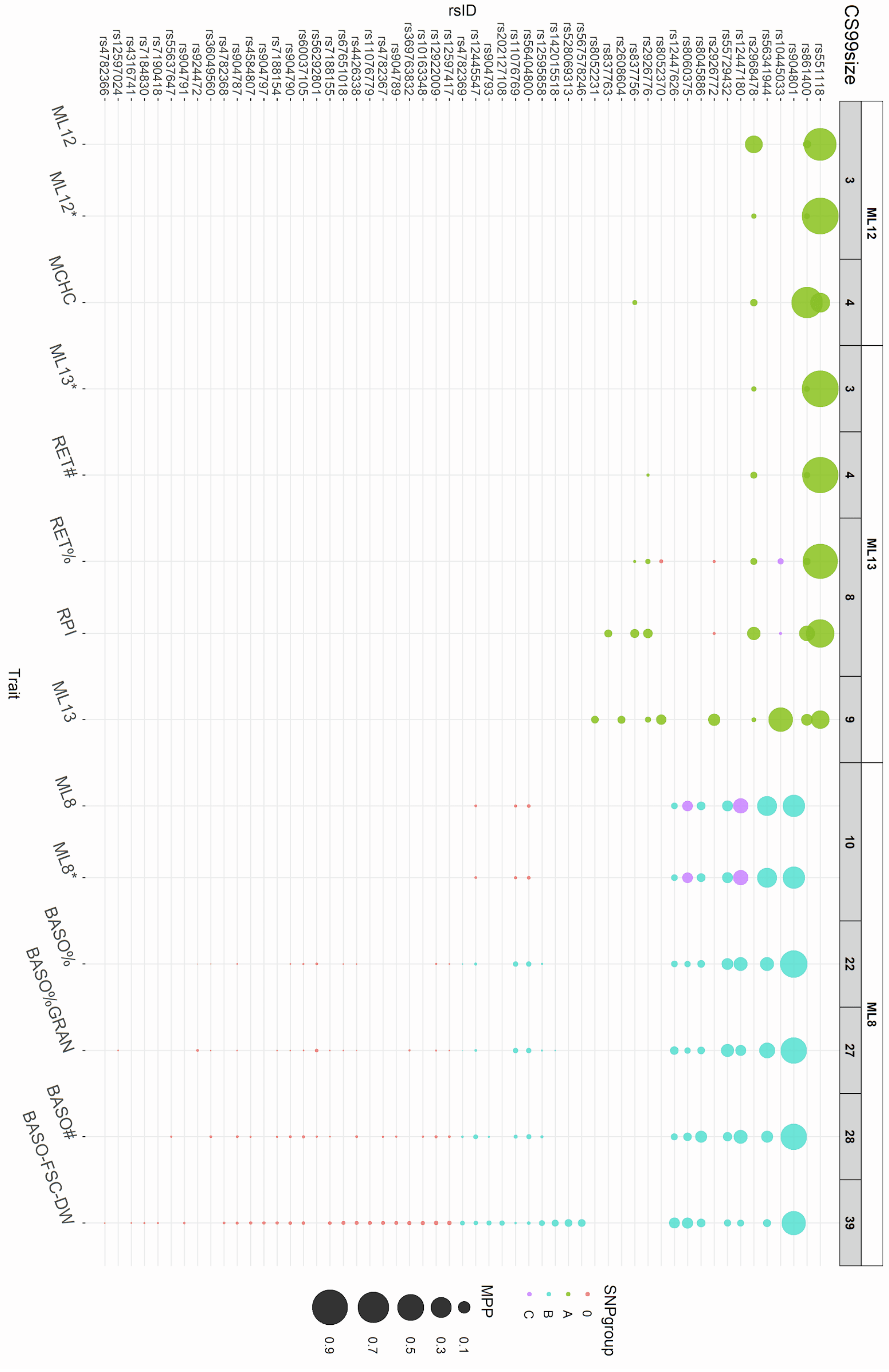

Figure S6. Latent and raw trait correlations in the *PIEZO1* region show high correlations amongst each latent trait with their linked raw traits, and amongst raw traits having a common latent trait. Related to STAR Methods. ML8 contributes to four basophil-related traits, ML12 contributes to MCHC, and ML13 contributes to three reticulocyte traits, as indicated by the correlation blocks.

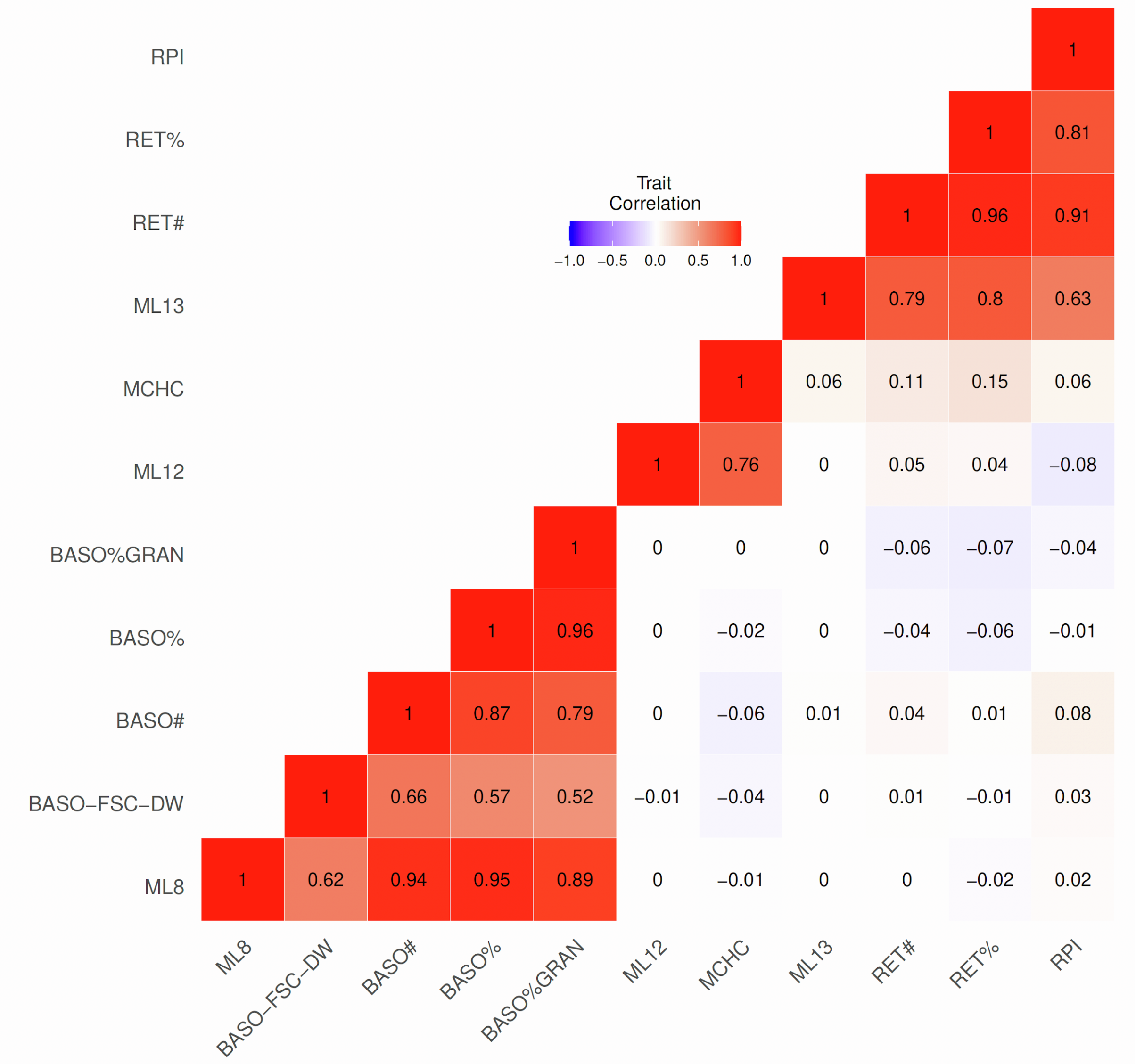

Figure S7. Latent and raw trait correlations in the *TMCC2* region show high correlations amongst each latent trait with their linked raw traits, and amongst raw traits having a common latent trait. Related to STAR Methods. ML5 contributes to nine platelet-related traits, and ML13 contributes to three basophil-related traits, as indicated by the correlation blocks.

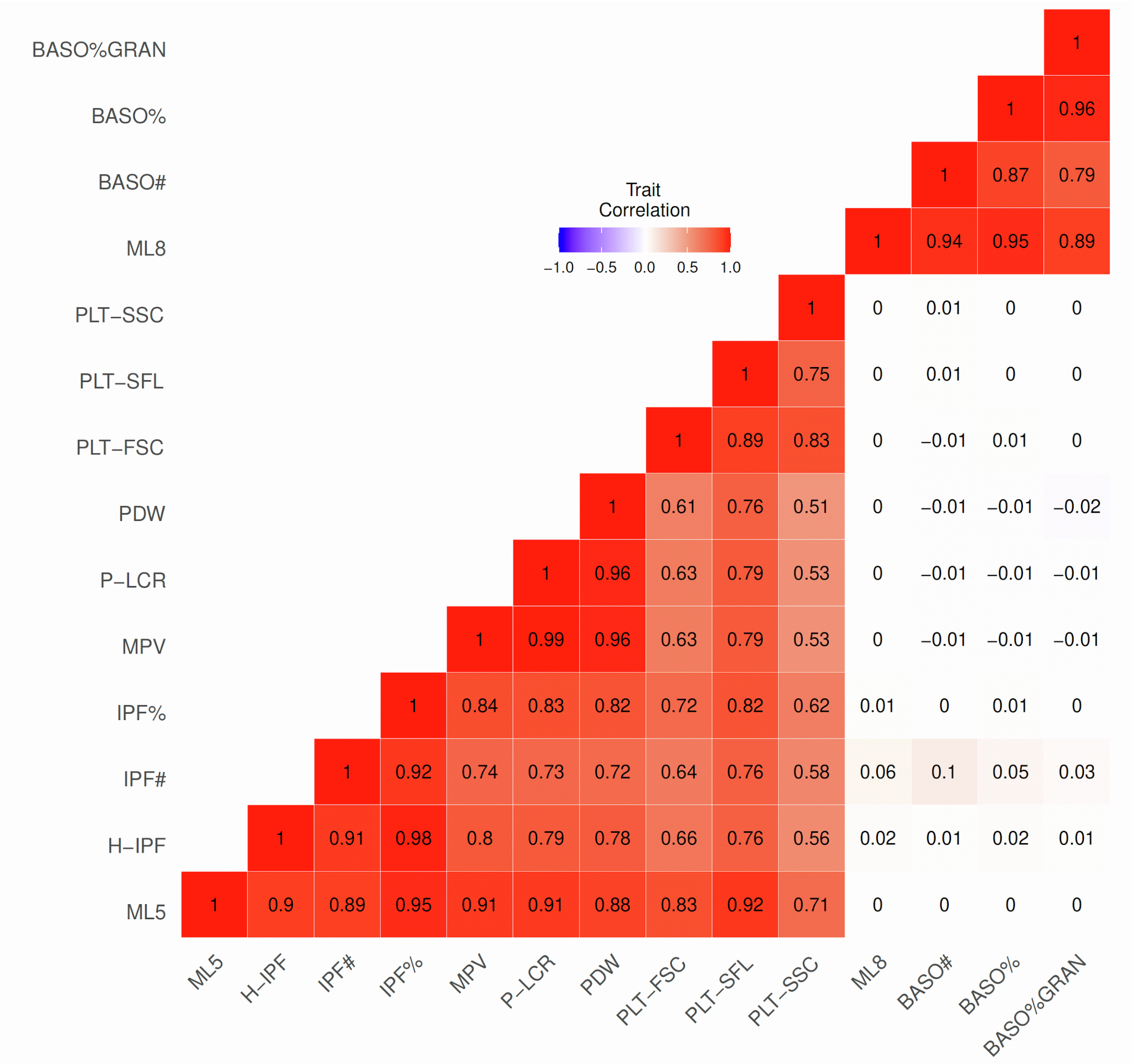

Figure S8. In the *TMCC2* region, the 99% credible sets (CS99) for raw traits become more refined for latent single and multi-trait fine-mapping with identical results between single-trait and multi-trait fine-mapping because of no shared causal variants. Related to STAR Methods. Variants belonging to at least one CS99 are listed at the left axis, labelled rsID. The CS99 for latent traits from single-trait fine-mapping are shown by the latent trait name, e.g. ML5. The CS99 for latent traits from multi-trait fine-mapping are shown by the latent trait name with an asterisk, e.g. ML5*. The coloured dots indicate which variants are members of the CS99 for the listed trait, where the size of each dot is proportional to the marginal posterior probability (MPP) of causality. Dot colours coincide with SNP groups, which are groups of variants in high LD ($r^2 > 0.8$) and with MPP > 0.01 as estimated by the fine-mapping method. Boxes indicate latent and raw traits that are related to each other and the CS99 sizes are provided for each trait, listed by increasing size, e.g. ML8 and ML8* both have a CS99 of size 12, BASO# and BASO% both have CS99 sizes of 18, and BASO%GRAN has 19 variants in its CS99 - these five traits are related to each other as indicated by the box.

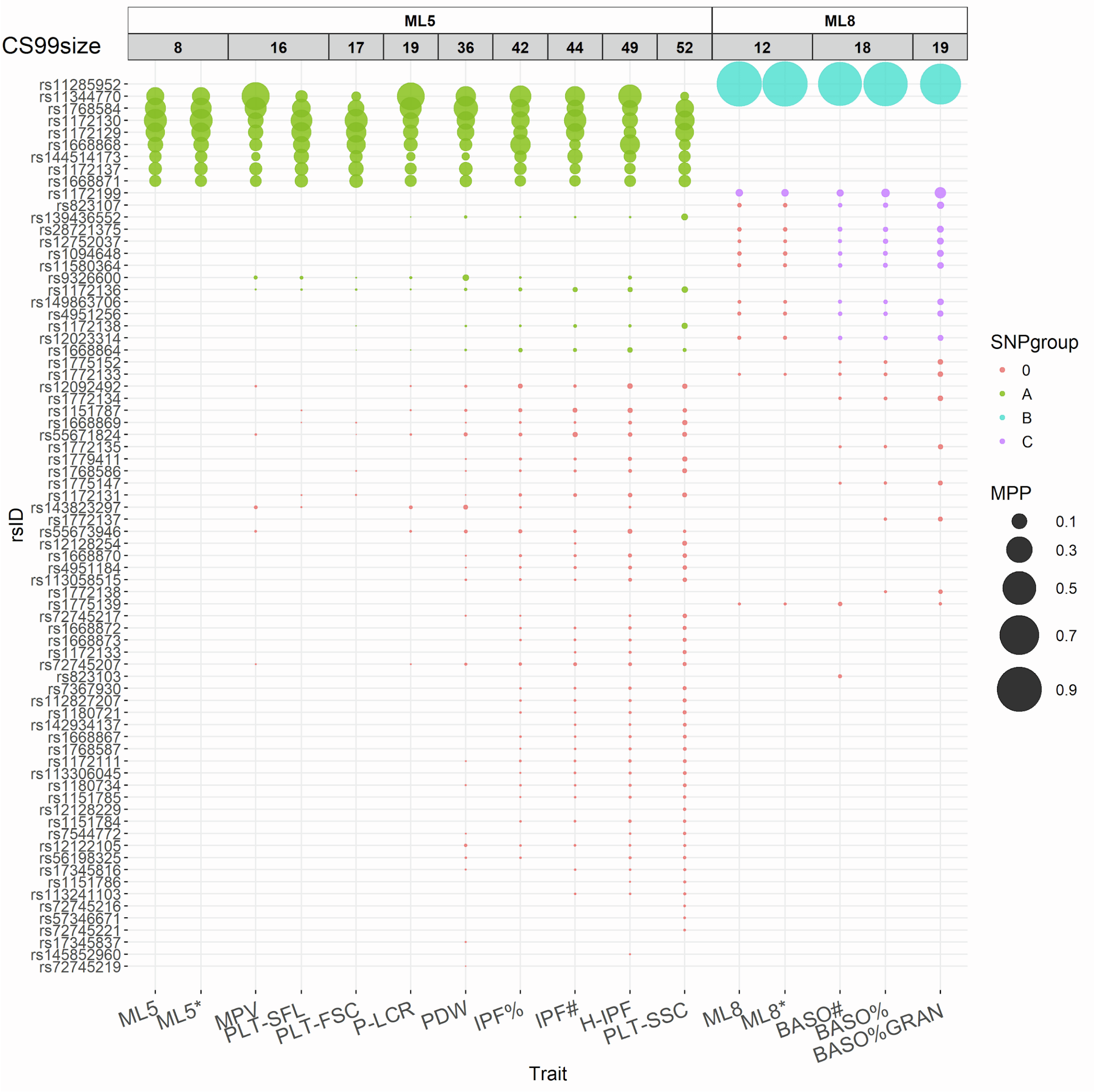

Figure S9. Selection of the number of latent factors for 99 blood cell traits using the correlation matrix of 43,059 individuals. Related to STAR Methods. This scree plot illustrates that 25 latent factors, as suggested by Horn's parallel method implemented in the "psych::fa.parallel" function in R, is optimal to explain the variability in 99 blood cell traits; the vertical line is at 25 latent factors. The blue line indicates the eigenvalues calculated in the observed data and the red dashed line corresponds to those calculated in random (noisy) data sets. The selection of 25 factors is the point where the observed data eigenvalues are smaller than those in the random data.

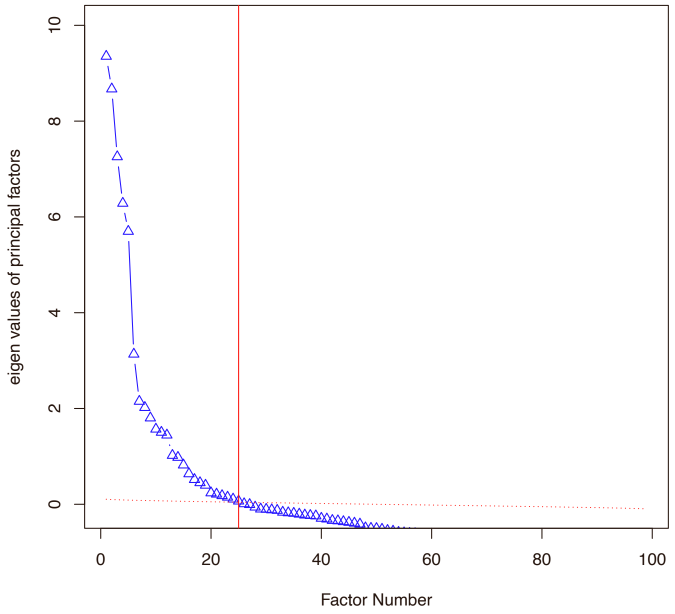

Figure S10. Comparison of latent factor contributions for 99 blood cell traits obtained from 18,310 individuals with complete data and from the correlation matrix of 43,059 individuals in the INTERVAL study. Related to STAR Methods. Each colour represents a single latent factor and its contributions to each of the 99 blood cell traits. This illustrates concordance between the latent factors obtained from the two analysis approaches.

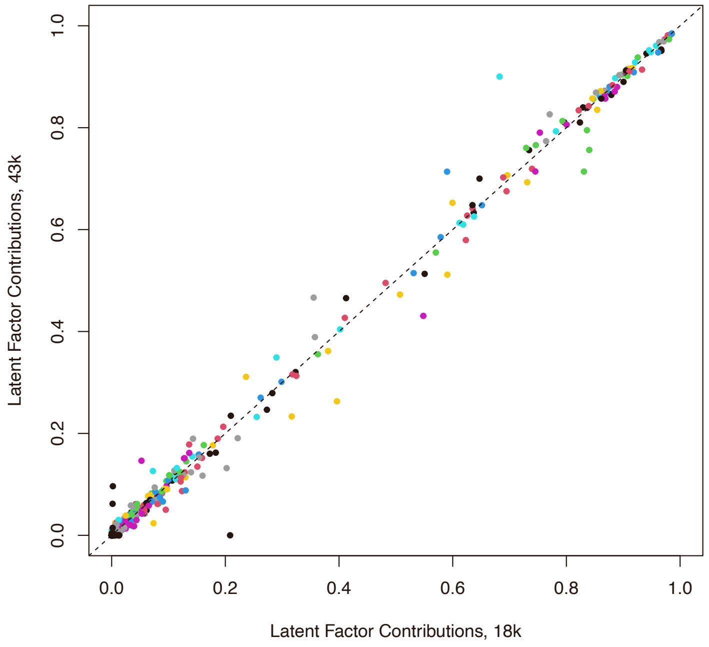

Figure S11. Selection of the number of latent factors for 184 metabolic traits in 40,849 individuals. Related to STAR Methods. This scree plot illustrates that 21 latent factors, as suggested by Horn's parallel method implemented in the "psych::fa.parallel" function in R, is optimal to explain the variability in 184 metabolic traits; the vertical line is at 21 latent factors. The blue line indicates the eigenvalues calculated in the observed data and the red dashed line corresponds to those calculated in random (noisy) data sets. The selection of 21 factors is the point where the observed data eigenvalues are smaller than those in the random data.

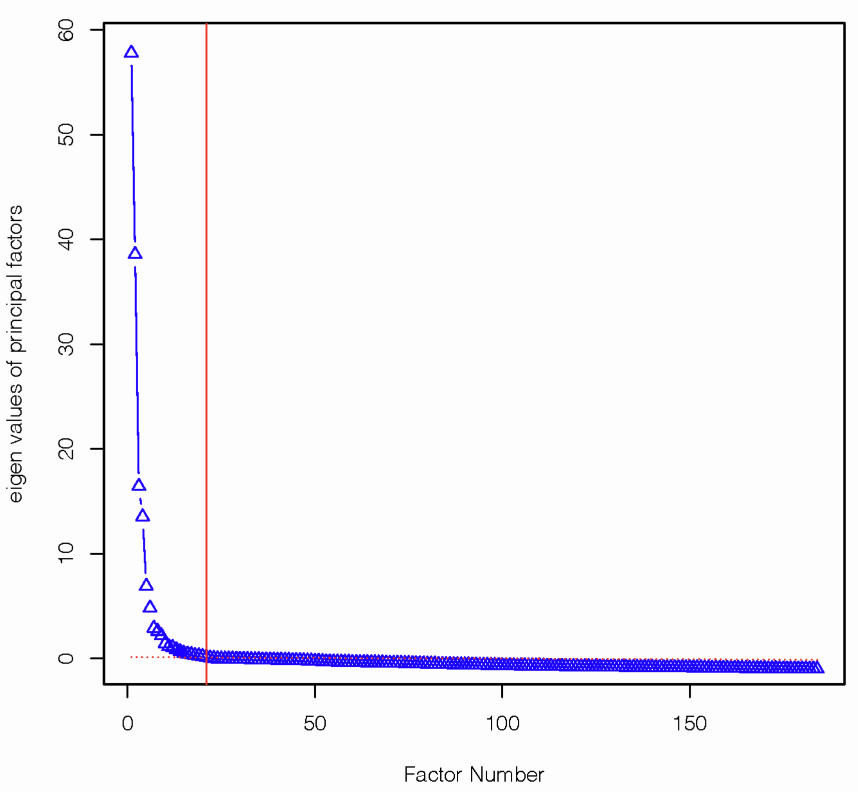

Supplement: Document S1. Figures S1–S11 [file mmc1.pdf]
